# Luteolin Attenuates Allergic Nasal Inflammation via Inhibition of Interleukin-4 in an Allergic Rhinitis Mouse Model and Peripheral Blood From Human Subjects With Allergic Rhinitis

**DOI:** 10.3389/fphar.2020.00291

**Published:** 2020-03-18

**Authors:** Kai-Li Liang, Sheng-Jie Yu, Wan-Chun Huang, Hung-Rong Yen

**Affiliations:** ^1^Department of Otolaryngology, Taichung Veterans General Hospital, Taichung, Taiwan; ^2^School of Medicine, Chung Shan Medical University, Taichung, Taiwan; ^3^Faculty of Medicine, National Yang-Ming University, Taipei, Taiwan; ^4^Department of Medical Education and Research, Kaohsiung Veterans General Hospital, Kaohsiung, Taiwan; ^5^Department of Medical Research, Research Center for Traditional Chinese Medicine, China Medicine University Hospital, Taichung, Taiwan; ^6^Department of Chinese Medicine, China Medicine University Hospital, Taichung, Taiwan; ^7^Chinese Medicine Research Center, China Medical University, Taichung, Taiwan; ^8^School of Chinese Medicine, College of Chinese Medicine, China Medical University, Taichung, Taiwan; ^9^Department of Biotechnology, Asia University, Taichung, Taiwan

**Keywords:** allergic rhinitis, IL-4, luteolin, traditional Chinese medicine, type 2 helper T cells

## Abstract

**Objectives:** Luteolin is the active component of *Perilla frutescens*, an herb for the treatment of allergy in Asia. In this study, we aimed to investigate the effects and mechanisms of luteolin treatment.

**Methods:** BALB/c mice sensitized with house dust mite (HDM) to induce allergic rhinitis (AR), and treated with dexamethasone or luteolin. In addition, mononuclear cells from peripheral blood (PBMC) of AR patients were co-cultured with dexamethasone or luteolin, and were re-stimulated with HDM.

**Results:** Luteolin-treated mice had decreased allergic symptoms, and serum HDM-specific IgE when compared to the untreated group. Flow cytometric analyses of splenocytes and nasal lymphoid tissues from AR mice found that luteolin decreased CD4+ IL-4-secreting T cells when compared to those from vehicle treated AR mice. Histopathology sections showed reduced infiltration of eosinophils and decreased mucus secretion of mouse nasal epithelium. In the *in vitro* study, the results showed that luteolin reduced the percentage of CD4+ IL-4-secreting splenocytes expression was through reducing expression of pSTAT6 and GATA3. PBMCs from AR patients pretreated with luteolin could decrease percentage of CD4+ IL-4-secreting cells.

**Conclusion:** Our study identified that luteolin attenuates allergic nasal inflammation via inhibition of IL-4 production, which supports the potential pharmaceutical application of luteolin treatment for AR.

## Introduction

Allergic rhinitis (AR) is one of the most common chronic diseases affecting both children and adults (Mattos et al., 2011). AR is defined as an immunoglobulin E (IgE)-mediated inflammatory response of the nasal mucosa. The nasal inflammation results in symptoms of itchiness, sneezing, rhinorrhea, and nasal congestion (Bousquet et al., 2008). AR can strongly impact quality of life and generate high economic and medical burden (Meltzer and Bukstein, 2001; Blaiss, 2010). Pharmacotherapy is the mainstream treatment of AR. Immunotherapy provides an additional choice for patients with AR who have inadequate responses to pharmacotherapy. Both pharmacotherapy and immunotherapy are effective treatments with strong supporting evidence. Nevertheless, many patients with allergic disorders turn to complementary therapies due to concerns about the side effects of long-term treatment. In our previous study, we screened one million randomly sampled beneficiaries in Taiwan's National Health Insurance Research Database and found 63.11% of pediatric AR patients had used traditional Chinese medicine (TCM) (Yen et al., 2015).

*Perilla frutescens* leaf is an herb of the Lamiaceae family. It is a popular herb for treatment of allergic diseases including AR and bronchial asthma in Asian countries. The main constituents of Perilla include flavonoids, saponins, and polysaccharides (Makino et al., 2003; Seo and Baek, 2009; Liu et al., 2013). Luteolin, a flavonoid, is believed to be the active anti-allergic component of *Perilla frutescens* (Ueda et al., 2002; Makino et al., 2003; Jeon et al., 2014). Ueda et al. (2002) found 8 mg luteolin can be isolated from 5.92 g of perilla leaf extract. Natsume et al. (2006) reported that the mean concentration of luteolin in Perilla leaf was 0.039 mg/g. Nevertheless, luteolin amount in Perilla leaf varies due to seasonal, geographic, or processing differences. Except for Perilla leaves, luteolin had been identified in many edible plants including carrots, peppers, celery, olive oil, thyme, rosemary, lettuce, turnip, and cucumber (López-Lázaro, 2009).

A recent study found that Perilla-derived methoxyflavanone effectively reduced the IgE-mediated histamine release from rat basophilic leukemia cells (Kamei et al., 2017). In another study, luteolin reduced cellular infiltration in pulmonary parenchyma and nasal tissue, as well as inflammatory cells in bronchoalveolar lavage fluid in a murine model of allergic asthma and rhinitis (Jang et al., 2017). Shen et al. (2016) found that luteolin could attenuate airway mucus overproduction in allergic mice. Another study found that luteolin demonstrated a regulatory effect on airway inflammation in an asthmatic rat model (Zeng et al., 2014). Nevertheless, the possible mechanisms of the immunomodulatory effects of luteolin have not been explored in detail by previous studies. Kritas et al. (2013) proposed that the anti-allergic effect of luteolin was via inhibition of mast cell activation. Kang et al. reported that luteolin exerted a regulatory effect on mast cell-mediated inflammation by suppression of several pro-inflammatory cytokines (Kang et al., 2010). Lee et al. found that luteolin could inhibit mucus overproduction of airway epithelial cells (Lee et al., 2015). While most of the researches focus on the effects of luteolin on airway epithelial cells and innate immune response, studies on its effect on type 2 helper T (T_H_2) cells was lacking. We proposed that luteolin inhibits T_H_2 inflammation thereby exerting anti-allergic effects. The purpose of this study was to evaluate the effect of luteolin on inhibition of T_H_2 inflammatory airway responses, in an AR animal model and in the mononuclear cells of peripheral blood (PBMC) of AR patients.

## Materials and Methods

### Allergic Rhinitis Mouse Model

The Institutional Animal Care and Use Committee of China Medical University approved the experimental animal protocol of the study. All animals were maintained and treated in accordance with the Principles of Laboratory Animal Care formulated by the National Society for Medical Research and the Guide for the Care and Use of Laboratory Animals prepared by the Institute of Laboratory Animal Resources, National Research Council, and published by the National Academy Press. Female BALB/c mice at 6–8 weeks of age were obtained from the National Laboratory Animal Center in Taiwan. Experimental mice were sensitized with intraperitoneal injection of 4 μg house dust mite (HDM, Indoor Biotechnologies Ltd, Cardiff, UK) mixed with 40 μg aluminum hydroxide gel adjuvant (Invitrogen, San Diego, CA) on days 0, 7, and 14, and received intranasal challenge with 4 μg HDM from days 22 to 26 (Kim et al., 2014).

### Grouping and Treatment Protocol for BALB/c Mice

BALB/c mice were assigned to blank, AR, low- (10 mg/kg) and high-dose (30 mg/kg) luteolin treatment (LO10 and LO30), and dexamethasone (Dex) treatment (1 mg/kg) groups. The blank group mice received null sensitization and null treatment (vehicle, dimethyl sulfoxide, DMSO), whereas the AR group mice received HDM sensitization and vehicle treatment (1% DMSO). The LO groups of mice received HDM sensitization and intraperitoneal injection of 10 mg/kg or 30 mg/kg luteolin (Sigma-Aldrich, St. Louis, MO, USA). Dex mice received HDM sensitization and intraperitoneal injection of dexamethasone treatment (1 mg/kg). Mice were sacrificed on day 27. Their blood, nasal tissues, and spleens were harvested for further analyses.

### Assessment of Allergic Rhinitis Symptoms

Allergic symptoms were assessed after the last intranasal challenge using a 10-min video recording of the mice. Frequency of nasal scratching and sneezing was calculated by reviewing the video in a blinded method. Two independent reviewers who were unaware of the grouping watched the videos and measured the times of nasal scratches and sneezes. The mean frequency of each individual mouse was then calculated (Supplemental Video 1).

### Assessment of Serum Immunoglobulin Levels

Sera were used for measurement of immunoglobulin E (IgE) and IgG levels with commercial mouse IgE and IgG isotype-specific enzyme–linked immunosorbent assay (ELISA) according to manufacturer's instructions (BD Pharmingen, San Diego, CA, USA). In brief, the ELISA plates were coated with capture antibodies. The pre-diluted serum samples and standards were added. After adequate incubation and washing, streptavidin-HRP and substrate solution was added. Then the ELISA plate was read at 450 nm to analyze the data.

### Histopathological Analyses of Nasal Tissues

Mice nasal tissues were fixed overnight in 10% neutral buffered formalin. Following fixation, the tissues were embedded in paraffin, cut into 5 μm sections and mounted on poly-L-lysine-coated glass slides. Tissue sections were stained with hematoxylin and eosin (H&E) and periodic acid-Schiff (PAS stain kit; ScyTek Laboratories, Inc., Logan, UT, USA) according to the manufacturer's instruction.

### Effects of Luteolin on Cytokine Production by Mouse Splenocytes and Nasal-Associated Lymphoid Tissue (NALT)

After sacrificing the mice, their spleens were harvested and sliced into pieces. The sliced spleen was pressed, washed, centrifuged and re-suspended in PBS with a concentration of 1 × 10^6^ cells per ml. Similarly, cells harvested from NALT of experimental mice were also collected. Cells were re-stimulated with PMA (50 ng/mL) and ionomycin (500 ng/mL) with GolgiStop (BD Biosciences) for 5 h. Surface staining with Pacific Blue anti-mouse CD4 antibody (BD Pharmingen, San Diego, CA, USA) was performed. Then cells were fixed and permeabilized (BD Cytofix/Cytoperm Kit, BD Bioscience, San Jose, CA, USA) followed by staining for intracellular cytokines. The following reagents were used: PerCP-Cy5.5 anti-mouse interferon-γ (IFN-γ, BD Pharmingen, San Diego, CA, USA), PE anti-mouse IL-4 (BioLegend, San Diego, CA, USA), FITC anti-mouse IL-17A (BioLegend, San Diego, CA, USA) antibodies. For the intracellular staining of FOXP3, we used the Foxp3/Transcription Factor Staining Buffer Set and FITC anti-mouse Foxp3 antibody (eBioscience, San Diego, CA, USA) according to the manufacturer's protocol. Flow cytometric acquisition was performed on the BD FACSVerse flow cytometer. Data were analyzed with FlowJo software (Tree Star, Ashland, OR, USA).

### Effects of Luteolin on Inhibition of HDM- Induced Cytokine Production

Mouse spleens were sliced and disrupted to obtain single cell suspensions. Cells were seeded on 96-well plates and then re-simulated with HDM (2 μg/ml) for 48 h. The supernatant was collected for detection of cytokines (Milliplex® Map mouse high-sensitivity T cell panel, based on Luminex®/xMAP® technology).

### Cells Purification and Treatment

BALB/c mice spleens were disrupted to obtain single cell suspensions. CD4+ T cells or naïve CD4+ T cells (CD4+CD44^low^CD62L^high^) were isolated by the EasySep Mouse CD4+ T Cell Isolation Kit or EasySep Mouse naïve CD4+ T Cell Isolation Kit (STEMCELL Technologies, Vancouver, British Columbia, Canada). Then the enriched CD4+ cell suspensions were plated with a concentration of 1 × 10^5^ cells per well. Culture medium for T_H_2 polarization contained Iscove's Modified Dulbecco's Medium (IMDM) supplemented with 5% FBS, 1% Penicillin/streptomycin solution, 50 μM 2-mercaptoethanol, 0.1 mM non-essential amino acid, 0.1 mM sodium pyruvate (all supplied by Gibco BRL, Grand Island, NY, USA), 100 ng/ml IL-4 (PeproTech, Rocky Hill, NJ, USA), and 10 ng/ml anti-IFN-γ neutralizing antibody (BioLegend, San Diego, CA, USA). Cells were then activated with plate-bound 5 μg/ml anti-CD3 and soluble 1 μg/ml anti-CD28 monoclonal antibodies (both from BioLegend, San Diego, CA, USA) for polyclonal activation.

Serial concentrations of luteolin (0.5, 1, 2.5, 5, and 10 μg/ml) were co-cultured with the CD4+ cells for 18 h. T_H_2-skewing cytokines were added to the culture media using the aforementioned doses. Cells were cultured for 2 days and then transferred to new wells without the coated anti-CD3 monoclonal antibody for 1 day. On day 3, cells were washed and replenished with new culture media with 10 ng/ml anti-IFN-γ neutralizing antibody (BioLegend, San Diego, CA, USA). On day 5, cells were collected and re-stimulated for 5 h in presence of PMA (50 ng/ml) and ionomycin (500 ng/ml) with GolgiStop (BD Biosciences) for 5 h followed by intracellular staining. Flow cytometric acquisition was performed on the BD FACSVerse flow cytometer, and analyzed with FlowJo software (Tree Star, Ashland, OR, USA).

### Western Blotting

Naïve CD4+ cells were pretreated with serial concentrations of luteolin (0.5, 1, 2.5, 5, and 10 μg/ml) for 18 h and then stimulated with IL-4 (100 ng/ml) (PeproTech, Rocky Hill, NJ, USA) for 6 h. Cells were then collected, washed, and lysed. Protein concentrations of supernatant from cell lysate were measured with BCA Protein Assay (Pierce Chemical Co., Rockford, IL, USA). Equal amounts of proteins from samples were subjected to electrophoretic separation in 10% polyacrylamide gel, transferred to polyvinylidene difluoride membrane, and then immersed in blocking buffer for 1 h at room temperature. The membrane was incubated with primary antibodies of STAT6, phosphorylated STAT6, GATA3 (all supplied by Cell Signaling Technology, Beverly, MA, USA, and Actin (Millipore Billerica, MA, USA) overnight at 4°C. On the following day, the membranes were incubated with HRP-conjugated secondary antibody after repeated washing and reacted at room temperature for 1 h, followed by electrochemiluminescent detection (Millipore Billerica, MA, USA). The density of each protein band was scanned using ImageJ Software, version 1.46r (National Institutes of Health, Bethesda, MD) and compared by densitometry.

### Study Subjects

The study in human subjects was approved by the Research Ethics Committee of China Medical University & Hospital (CMUH106-REC1-031) and Taichung Veterans General Hospital (CE17344A). All research was performed in accordance with relevant research guidelines and regulations. Patients with AR caused by HDM and non-AR control subjects were recruited. Inform consents were obtained from all study subjects. HDM-induced AR patients all had a history of nasal symptoms including itchiness, rhinorrhea, or nasal obstruction in dusty environments. They were enrolled if their serum specific IgE for HMD > 0.35I U/ml. Control subjects had no rhinitis symptoms, their serum total IgE levels were < 100 IU/ml, and specific HDM IgE was < 0.35I U/ml (Immulite 2,000 Immunoassay system; Siemens Healthineers, Flanders, NJ, USA). Peripheral venous blood was collected from the study subjects. The PBMCs were isolated from the blood samples by centrifugation through Ficoll-Hypaque gradients (GE Healthcare Bio-Sciences, Uppsala, Sweden) according to the manufacturer's instructions. PBMCs with a concentration of 1 × 10^6^ per well were co-cultured with HDM (2 μg/ml) and serial concentrations of luteolin (1.25, 2.5, or 5 μg/ml) or 2 μg/ml dexamethasone (Sigma-Aldrich, St. Louis, MO, USA) in 10% FBS RPMI 1,640 medium (Gibco BRL, Grand Island, NY, USA). Culture medium was replaced on the 3rd day. On day 6, the cells were collected for intracellular staining. Supernatants of the culture medium were then analyzed using an ELISA kit (BD Pharmingen, San Diego, CA, USA). Reagents used for this experiment were: PC5 anti-human CD4, PE anti-human IL-4, and APC anti-human IFN-γ antibodies (all supplied by Beckman Coulter, Fullerton, CA, USA). Flow cytometric acquisition was performed on the BD FACSVerse flow cytometer, and analyzed with FlowJo software (Tree Star, Ashland, OR, USA).

### Statistical Analyses

The results of differences amongst AR, LO10, and LO30 mice, CD4+ cells co-cultured with HDM and serial concentrations of luteolin (0.5, 1, 2.5, 5, or 10 μg/ml), PBMCs co-cultured with HDM and serial concentrations of luteolin (1.25, 2.5, or 5 μg/ml) were performed by non-parametric Kruskal-Wallis test. Dunn's multiple comparisons test was used for *post hoc* examination of between-group differences. In addition, Mann-Whitney U test was used for analyses differences between AR vs. blank mice, AR vs. Dex mice, negative vs. positive control PBMCs (medium with or without HMD), and control vs. Dex co-culture PBMCs. Statistically significance was defined as a *P* < 0.05. Statistical analyses were performed using GraphPad Prism 6.0 (GraphPad Software, La Jolla, CA, USA).

## Results

### Luteolin Use Before House Dust Mite (HDM) Sensitization Could Reduce AR Symptoms

**Figure 1:**
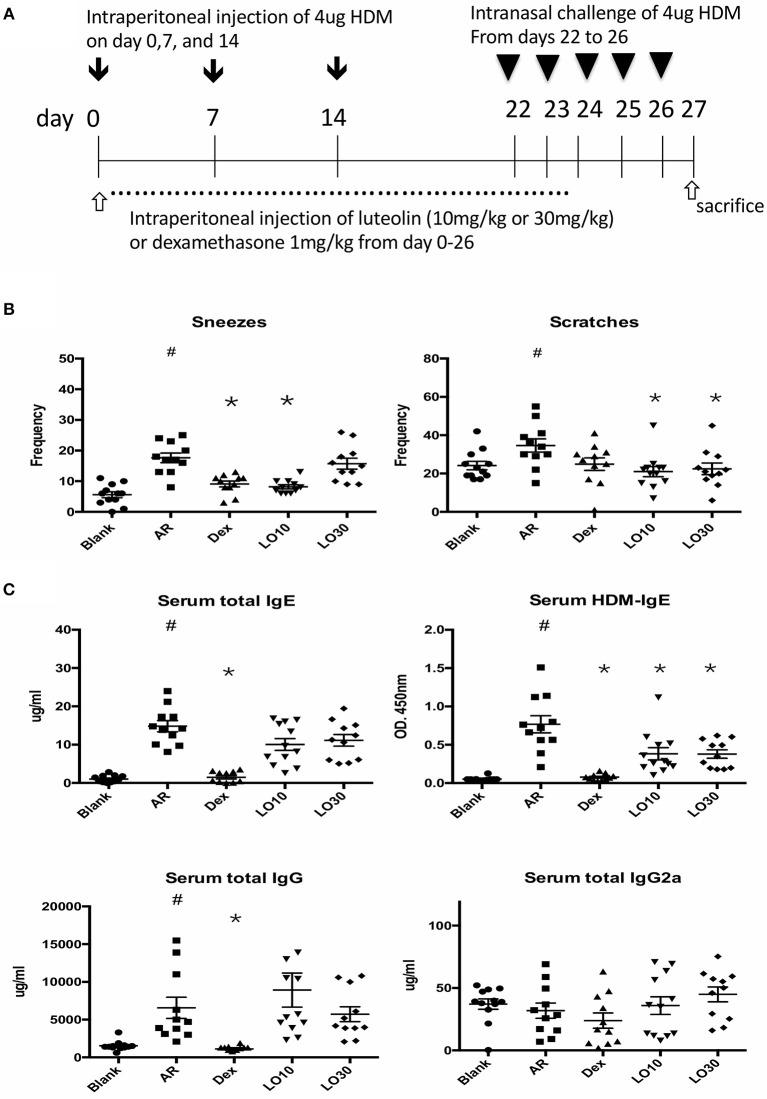
**(A)** Sensitization and treatment protocols for the experimental mice. **(B)** Nasal symptoms of allergic mice decreased after luteolin or dexamethasone treatment. **(C)** Levels of immunoglobulin (Ig) E and IgG of experimental mice. HDM = house dust mite; Blank = mice with null sensitization and treatment; AR = allergic mice with vehicle treatment; Dex = allergic mice treated with 1 mg/kg dexamethasone; LO10 = allergic mice treated with 10 mg/kg luteolin; LO30 = allergic mice treated with 30 mg/kg luteolin; ^#^*P* < 0.05 vs. blank group; *P < 0.05 vs. AR group.

HDM-sensitized mice (AR group) had significantly increased frequency of allergic symptoms including sneezing and nose scratching when compared with mice in the blank group (*P* < 0.001 and *P* = 0.025, respectively). AR mice also had significantly higher total and HDM-specific IgE compared to the blank group mice (both *P* < 0.0001). AR mice pre-treated with low-dose luteolin (10 mg/kg, LO10), high-dose luteolin (30 mg/kg, LO30), or dexamethasone (Dex) had decreased allergic symptoms when compared with AR mice without treatment (sneezing: AR vs. LO10, AR vs. Dex, *P* = 0.0003 and *P* = 0.0002, respectively; nose scratching: AR vs. LO10, AR vs. LO30, *P* = 0.0013 and *P* = 0.030, respectively). AR mice that received either luteolin or Dex treatment had significantly decreased HDM-specific IgE (AR vs. LO10, AR vs. LO30, AR vs. Dex, *P* = 0.009, 0.014, and < 0.0001, respectively) (Figure 1).

### Effects of Luteolin on Cytokines of Nasal Lymphoid Tissues (NALT) or Splenocytes From AR Mice

**Figure 2:**
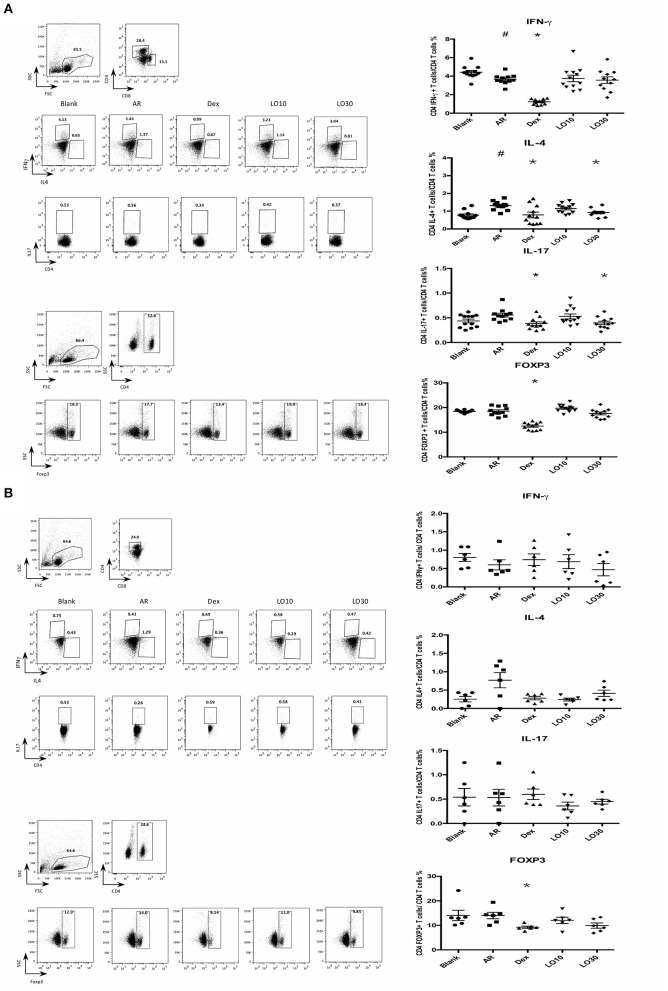
Cytokines and FOXP3 expression of CD4+ splenocytes **(A)** or nasal lymphoid tissues **(B)** from experimental mice. AR = allergic rhinitis group; Dex = allergic mice treated with 1 mg/kg dexamethasone; LO10 = allergic mice treated with 10 mg/kg luteolin; LO30 = allergic mice treated with 30 mg/kg luteolin; ^#^*P* < 0.05 vs. blank group; *P < 0.05 vs. AR group.

Flow cytometry was used to analyze splenocytes harvested from BALB/c mice: We found that the percentage of CD4+ IL-4-secreting T cells was significantly decreased in splenocytes from AR mice treated with dexamethasone compared to those of AR mice (P = 0.019). In addition, the percentages of CD4+IFN-γ-, CD4+ IL-17-secreting T cells, and CD4+ FoxP3+ T cells significantly decreased in AR mice treated with dexamethasone (*P* < 0.0001, *P* = 0.002, and *P* < 0.0001 vs. AR). The percentages of CD4+ IL-4-, CD4+ IL-17-secreting T cells decreased in splenocytes harvested from 30 mg/kg luteolin-treated AR mice as well (*P* = 0.0026 and *P* = 0.012 vs. AR) (Figure 2A).

Similar effect on IL-4 expression in NALT from mice treated with dexamethasone and luteolin compared to that of AR group mice (Figure 2B). Nevertheless, there were no significant between-group differences.

### Histological Examination of Nasal Tissues From AR Mice

**Figure 3:**
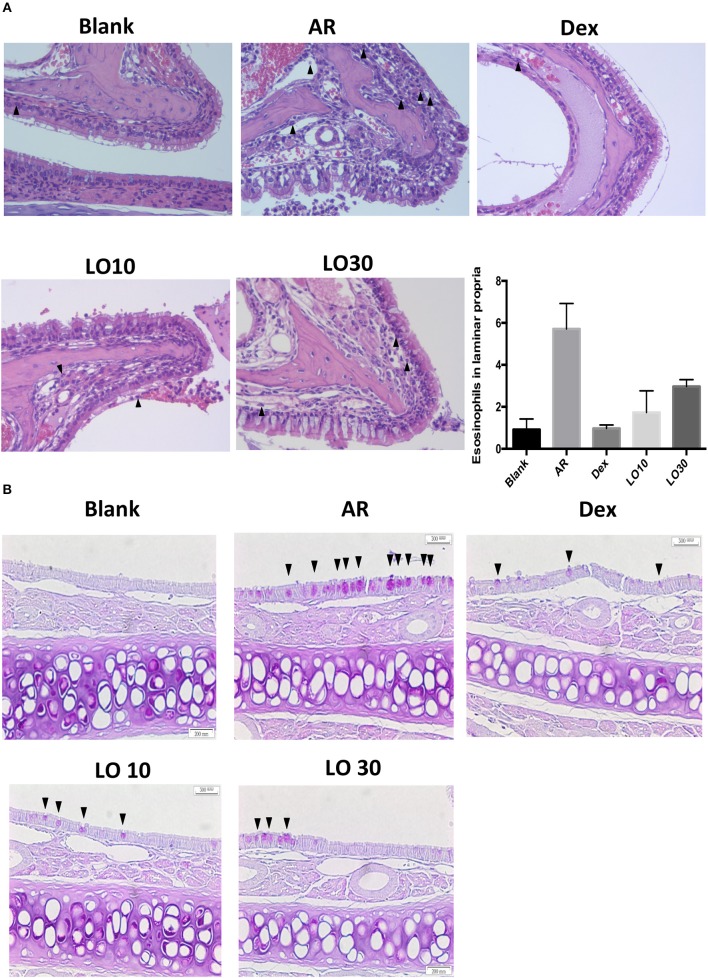
**(A)** Hematoxylin and eosin staining (400×) of tissue sections from experimental mice. The arrowheads mark the eosinophils in laminar propria. **(B)** Periodic acid-Schiff staining of the nasal mucosa from experimental mice (400×). The arrowheads mark the PAS staining cells.

Hematoxylin and Eosin (H&E) staining of nasal tissues from the allergic mice demonstrated increased immune cells infiltration. Allergic mice that received dexamethasone or luteolin treatment had less submucosal cellular infiltration and eosinophils in the laminar propria compared to AR mice (Figure 3A). Periodic acid-Schiff (PAS) staining of nasal mucosa was performed to evaluate mucus production and the results showed that increased mucus production was observed in nasal mucosa of the allergic mice and the mucus production obviously decreased after treatment (Figure 3B).

### Effects of Luteolin on Cytokines Production by HDM Stimulated Splenocytes From AR Mice

**Figure 4:**
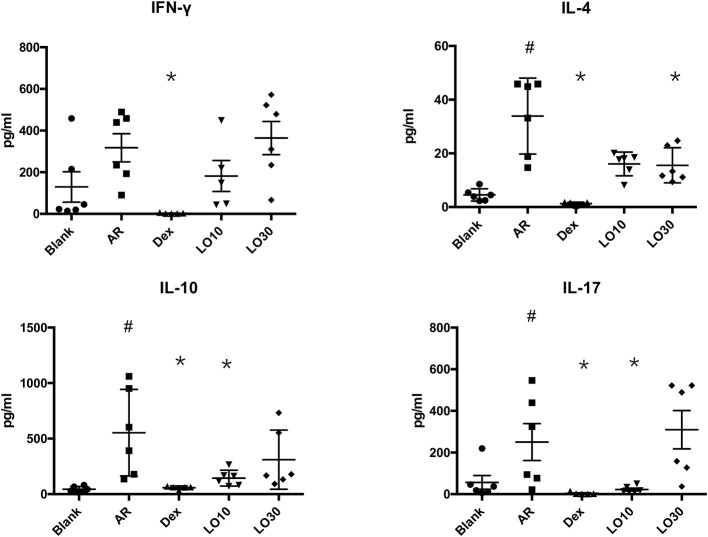
Levels of cytokines in the supernatants of culture medium from splenocytes harvested from experimental mice re-stimulated with house dust mite. ^#^*P* < 0.05 vs. blank group; *P < 0.05 vs. AR group.

Splenocytes harvested from each group of mice were re-stimulated with HDM for 48 h. The supernatant of culture media was analyzed by Luminex®. The results showed that IL-4 production significantly decreased in Dex and LO30 groups compared to that of AR group (*P* = 0.004 and *P* = 0.040, respectively). The expression of IL-10 and IL-17 significantly decreased in Dex and LO10 groups compared to those of AR group (AR vs. Dex: both *P* = 0.004; AR vs. LO10: both *P* = 0.029). Furthermore, there was significantly difference between AR and Dex groups in IFN-γ expression (*P* = 0.004) (Figure 4).

### Luteolin Treatment After HDM Sensitization Could Reduce AR Symptoms

**Figure 5:**
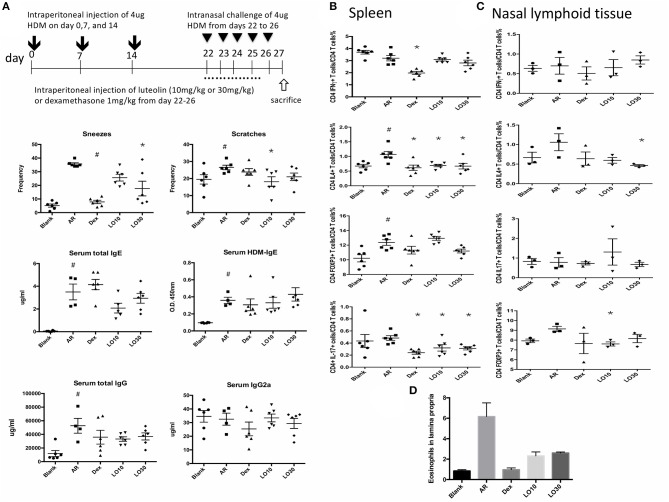
**(A)** Sensitization and treatment protocols for the experimental mice, nasal symptom and immunoglobulin levels. **(B,C)** Cytokines expression of CD4+ splenocytes and nasal lymphoid tissues. **(D)** Decreased eosinophil infiltration in Hematoxylin and eosin staining of tissue sections from experimental mice. AR = allergic rhinitis group; Dex = allergic mice treated with 1 mg/kg dexamethasone; LO10 = allergic mice treated with 10 mg/kg luteolin; LO30 = allergic mice treated with 30 mg/kg luteolin; ^#^*P* < 0.05 vs. blank group; *P < 0.05 vs. AR group.

The aforementioned results demonstrated that luteolin use before sensitization significantly ameliorated disease severity. We then evaluated the effect of luteolin effect after HDM sensitization on AR symptoms. In the frequencies of scratch and sneeze, treatment with LO10 and LO30 could significantly reduce the frequencies compared with AR group (sneeze: AR vs. LO30, *P* = 0.017; scratch: AR vs. LO10, *P* = 0.039). However, there were no significant differences in total IgE and HDM-IgE among luteolin groups and AR group. Neither total IgG nor IgG2a of luteolin groups were significantly different from those of AR group (Figure 5A). We used the flow cytometry to analyze splenocytes and NALT isolated from AR mice. The results showed that percentage of CD4+IL-4+ cells decreased after luteolin treatment (splenocytes: *P* = 0.046 and *P* = 0.015 for LO10 and LO30; NALT: *P* = 0.034 for LO30). The percentage of CD4+IL-17+ cells decreased after luteolin treatment as well (splenocytes: *P* = 0.043 and *P* = 0.02 for LO10 and LO30) (Figures 5B,C). Furthermore, eosinophils infiltration decreased in H&E staining of nasal tissue in luteolin treatment groups compared to that of AR group (Figure 5D).

### Luteolin Suppresses Th2 Cells Expression Through Inhibiting pSTAT6 and GATA3 Expression *in vitro*

**Figure 6:**
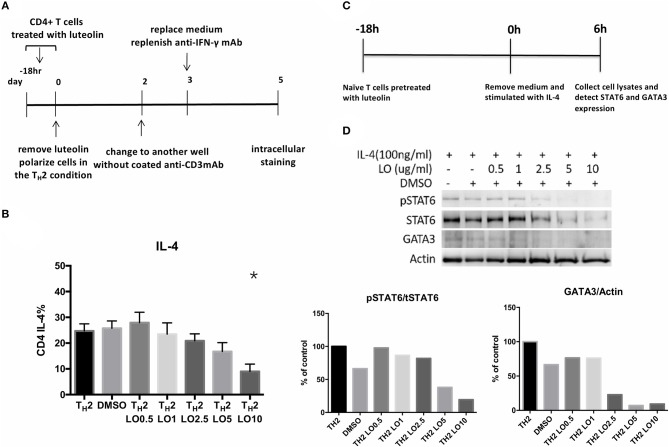
**(A)** Experimental protocols for evaluating the efficacy of luteolin on murine CD4+ cell polarization. **(B)** Pre-treatment of luteolin reduced the percentage of CD4+ IL-4-secreting cells. *P < 0.05 vs. T_H_2. **(C)** Experimental protocols for evaluating the efficacy of luteolin on murine naïve CD4+ cell polarization. **(D)** STAT6 and GATA3 protein expression of mouse naïve CD4+ cells were detected by western blot. pSTAT6 = phosphorylated (active) STAT6 protein; tSTAT6 = total STAT6 protein.

CD4+ splenocytes were isolated and then cultured with serial concentrations of luteolin (0.5, 1, 2.5, 5, 10 μg/ml) for 18 h (Figure 6A). T_H_2-skewing cytokines were then added into the culture media. Intracellular staining performed on the 5th day. A dose-responsive effect of luteolin in reducing the percentage of IL-4-secreting CD4+ cells was found (cultured without luteolin vs. with 10 μg/ml luteolin, *P* = 0.013) (Figure 6B). Naïve CD4+ cells isolated from BALB/c mice spleen were pretreated with serial concentrations of luteolin for 18 h and then were stimulated with IL-4 for 6 h (Figure 6C). The results showed that expression of pSTAT6 and GATA3 decreased after pretreated with luteolin. These data indicated that luteolin could reduce T_H_2 cells differentiation through inhibiting pSTAT6 and GATA3 expression (Figure 6D).

### Effects of Luteolin on Human Peripheral Blood Mononuclear Cells (PBMC)

**Figure 7:**
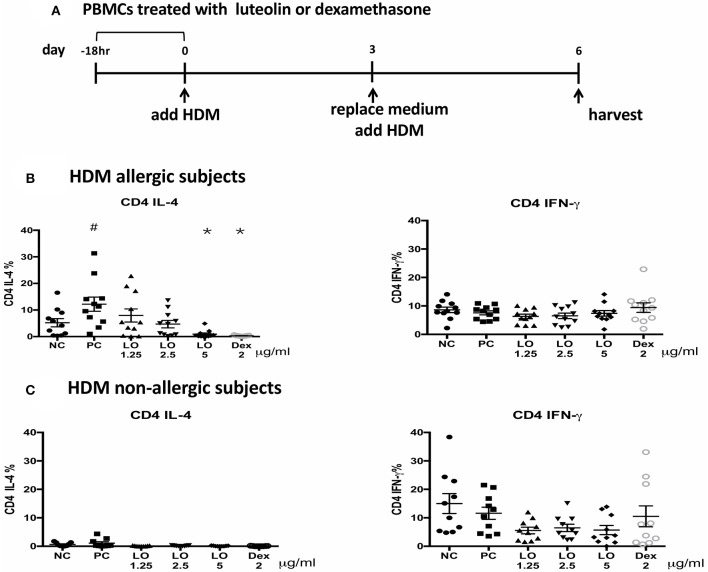
Luteolin inhibited CD4+IL-4+ cells expression of PBMCs isolated from HDM-AR or non-AR patients. **(A)** The protocol for human PBMCs pre-treatment with serial concentrations of luteolin and dexamethasone. Decreased percentage of CD4+ IL-4-secreting cells in PBMCs of **(B)** HDM allergic and **(C)** HDM non-allergic subjects after pretreatment with luteolin or dexamethasone. NC = negative control (medium without HDM); PC = positive control (medium with HDM); LO & Dex = pretreated with luteolin or dexamethasone, cultured in medium with HDM. ^#^*P* < 0.05 vs. NC group. *P < 0.05 vs. PC group.

A total of 16 subjects were enrolled in this study: 8 were HDM-allergic rhinitis patients and the others were controls. There were 5 males and 11 females with a mean age of 34.6 years. The mean total IgE level of allergic subjects was 356 (124–933) IU/ml, and HDM-IgE ranged from 9.29 to > 100 IU/ml. The mean total IgE for non-AR subjects was 24 (2.9–63.8) IU/ml, and HDM-IgE ranged from 0.1–0.288 IU/ml. PBMC from AR subjects cultured with HDM induced higher CD4+ IL-4+ cells compared to those from non-AR controls. After pretreatment with luteolin for 18 h, the percentage of CD4+ IL-4-secreting cells decreased by flow cytometric analyses (*P* = 0.0001 for luteolin 5 μg/ml vs. control) (Figure 7). These results suggested that the effects of luteolin on inhibiting T_H_2 cells expression could be found both in murine and human samples.

## Discussion

Perennial allergic rhinitis (AR) is a common allergic disorder in Taiwan. For moderate to severe perennial AR subjects, they usually require long-term medical treatment. Many AR patients therefore turn to complementary therapies to avoid possible side effects of prolonged anti-allergic medication. TCM is the most popular complementary treatment used for allergic disease in Asian countries (Man, 2009; Hon et al., 2015; Yonekura et al., 2017). According to our previous survey, about 34% patients who visited our department for rhinological disorders had received TCM treatment (Koo et al., 2014). Although there is evidence supporting the efficacy of TCM for treatment of AR, the methodology of these clinical trials was frequently inadequate. Furthermore, few studies have explored the treatment mechanisms of TCM. Currently, international treatment guidelines for AR do not support the use of complementary and alternative treatment including herbal medicine for AR (Meltzer and Bukstein, 2001; Passalacqua et al., 2006). As a significant proportion of allergic patients use TCM, there is an unmet need to investigate treatment efficacy and safety of TCM for the treatment of AR.

Various herbal formulas have been prescribed for the treatment of AR. *Perilla frutescens* is a commonly used herb for treatment of allergic diseases. The active component of *Perilla frutescens*, luteolin, is believed to have anti-allergic, anti-inflammatory, and anti-oxidative effect (Seelinger et al., 2008; Lee et al., 2010, 2015; Shen et al., 2016; Jang et al., 2017; Kamei et al., 2017; Kim and Yun, 2017; Nunes et al., 2017). Several possible mechanisms of the anti-allergic effects of luteolin have been proposed by previous studies and these include inhibition of pro-inflammatory cytokines, reduction of IgE production, and reduction of mucin production (Kritas et al., 2013; Lee et al., 2015; Shen et al., 2016; Jang et al., 2017). In the present study, we investigated the effects and mechanism of luteolin in a murine AR model. Our results found that luteolin effectively reduced the allergic symptoms, serum IgE, and mucus production, as well as local and systemic IL-4 production of allergic experimental mice. Dexamethasone was used as a positive control in this study because of its strong anti-allergic effect. In our results, luteolin demonstrated comparative anti-allergic effect as dexamethasone.

**Figure 8:**
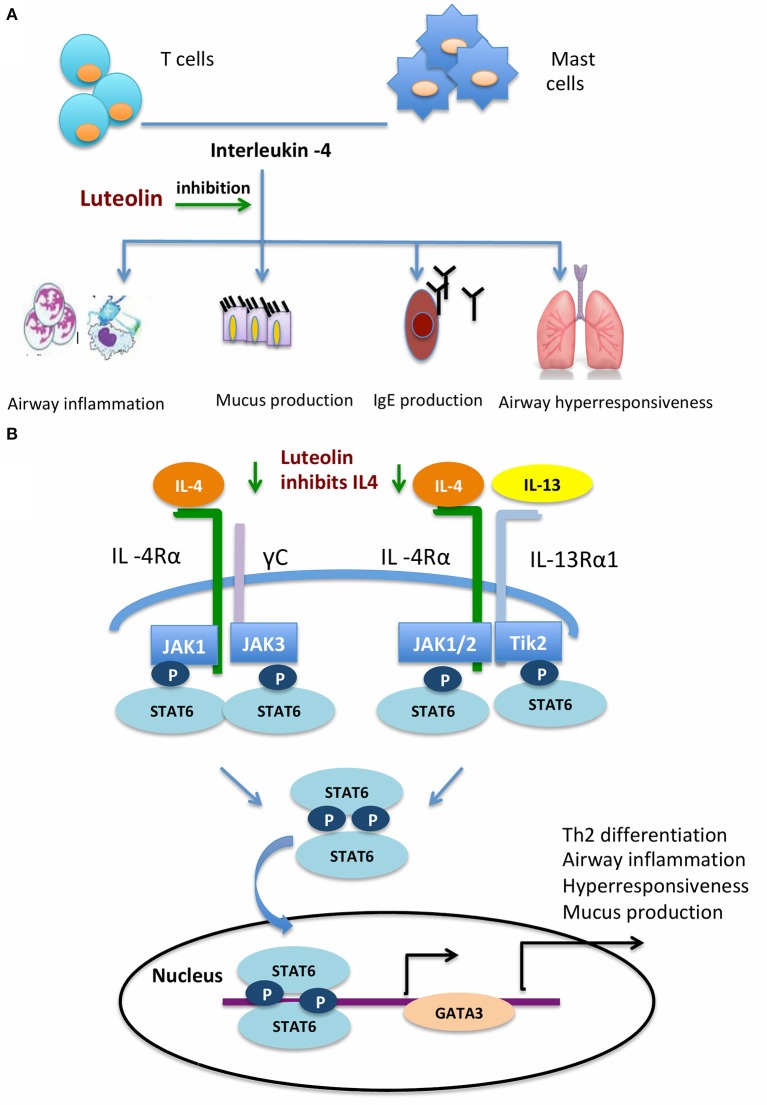
Schematic diagram depicting how luteolin inhibits airway allergic responses. **(A)** Luteolin inhibits IL-4 induced airway inflammatory responses. **(B)** Luteolin acts through the IL-4/STAT6/GATA3 signaling pathway.

IL-4 is an important cytokine produced by mast cells and lymphocytes (McLeod et al., 2015; Oeser et al., 2015). IL-4 plays several important roles in the pathogenesis of allergic asthma and allergic rhinitis (Zissler et al., 2015, 2016; Chai et al., 2017). Mucus hyper-production is a main allergic reaction. IL-4 could enhance the effect of histamine-induced mucus hyper-secretion in allergic responses (Babina et al., 2016; Kang et al., 2017). IL-4 promotes immunoglobulin production and isotype switching by B cells (Wu and Scheerens, 2014; Robinson et al., 2017). It also promotes production of IL-13, another important cytokine in allergic reactions, in mast cells (Toru et al., 1998). Compared with IL-13, IL-4 is more potent in mast cell proliferation and differentiation (Nilsson and Nilsson, 1995; Sherman et al., 2002). Both IL-4 and IL-13 are key cytokines in the activation of goblet cells and smooth muscle cells, and recruitment of more effector cells including eosinophils (Walford and Doherty, 2013) (Figure 8A). After binding with their receptors, IL-4 and IL-13 activate of Janus Kinase (JAK), which consequently phosphorylates STAT6 to the biologically active form (pSTAT6). STAT6 mediates the important functions of IL-4 and IL-13. STAT6 knockout mice did not respond to lL-4 and IL-13, failed to produce IgE, and developed airway hyperresponsiveness after allergen exposure (Tomkinson et al., 1999; Kim et al., 2006). Phosphorylated STAT6 then enhances the GATA-3 transcription factor, further activates T_H_2 cell differentiation, and promotes the secretion of IL-4, IL-5, and IL-13 (Figure 8B). In the present study, we found that the pSTAT6 and GATA3 were reduced after naïve CD4+ cells were pretreated with luteolin. The attenuation of nasal allergic symptoms by luteolin might act through inhibition of the IL-4/STAT6/GATA3 signaling pathway. It has to be mentioned that the activation of GATA3 might further promote the secretion of IL-4 from T_H_2 cells. Therefore, inhibition of this loop may provide an opportunity to the treatment of allergic disorders.

AR results from an imbalance between T_H_1- and T_H_2- immune responses. In our results, we found that CD4+ lymphocytes co-cultured with luteolin reduced the ratio of T_H_2 polarization. Furthermore, we found that serial concentrations of luteolin demonstrated a dose-responsiveness relationship in the inhibition of naïve CD4+ T cell skewing to T_H_2 cells. This indicates that luteolin is a promising agent for the prevention of allergic airway diseases. In addition, splenocytes from luteolin-treated allergic mice reduced IL-4 secretion after allergen re-stimulation. Luteolin could inhibit allergic responses during allergen exposure after sensitization. In this study, we also examined the effect of luteolin with PMBC collected from HDM-allergic subjects. The *in vitro* experiments in human blood samples confirmed that luteolin inhibits production of T_H_2 cytokines, IL-4. Further clinical studies to translate these results into pharmacotherapeutic intervention with luteolin in clinical AR patients are needed in the future.

There are some limitations in this study. First, although we found luteolin attenuated nasal allergic symptoms partly by inhibition of the IL-4-STAT6-GATA3 axis, other pharmacological actions that contribute to the mitigation of AR symptoms, such as anti-oxidative properties that have been found in many naturally occurring flavonoids cannot be completely ruled out. Second, the administration route of luteolin was via intraperitoneal injection in our study, further determination of the intranasal efficacy is warranted in the future experiments. Lastly, we found that luteolin was effective in AR mice and in the *in vitro* study of HDM-stimulated human PBMC. Nevertheless, further clinical trials are necessary to prove the clinical benefits.

Our results from mice experiments revealed that luteolin was effective at amelioration of allergic symptoms, serum IgE, and airway mucus production by reducing IL-4 secretion. Luteolin inhibited murine T_H_2 polarization as well as T_H_2 cytokines production in human PBMC. Therefore, luteolin is a promising candidate for the new drug development for new AR therapies.

## Data Availability Statement

All datasets generated for this study are included in the article/Supplementary Material.

## Ethics Statement

The studies involving human participants were reviewed and approved by the Research Ethics Committee of China Medical University & Hospital (CMUH106-REC1-031) and Taichung Veterans General Hospital (CE17344A). The patients/participants provided their written informed consent to participate in this study. The animal study was reviewed and approved by The Institutional Animal Care and Use Committee of China Medical University.

## Author Contributions

K-LL and H-RY conceptualized the study. S-JY, W-CH, and H-RY contributed to the animal experiments. K-LL, S-JY, and W-CH contributed to the recruitment of human subjects and performed experiments. K-LL, S-JY, W-CH, and H-RY contributed to the interpretation of experimental results. K-LL drafted the manuscript. K-LL and H-RY finalized the manuscript.

### Conflict of Interest

The authors declare that the research was conducted in the absence of any commercial or financial relationships that could be construed as a potential conflict of interest.

## Supplementary Material

Video S1 Nasal scratching and sneezing of the allergic mouse.
